# Predictive factors of disease-free survival after complete pathological response to neoadjuvant radiotherapy for rectal adenocarcinoma: retrospective case series

**DOI:** 10.1186/s12885-019-6239-3

**Published:** 2019-10-28

**Authors:** Amine Souadka, Mohammed Anass Majbar, Amine Benkabbou, Badr Serji, Tarik Souiki, Sidi Mohammed Bouchentouf, Mourad Abid, Basma El Khannousi, Tijani El Harroudi, Hadj Omar El Malki, Mohammed Raiss, Lahsen Ifrine, Khalid Mazaz, Aziz Zentar, Raouf Mohsine, Abdelilah Souadka, Abdelkader Belkouchi, Mohammed Ahallat, Abdelmalek Hrora, on behalf of the Moroccan Society of Surgery

**Affiliations:** 1 Surgical Oncology Department, National Institute of Oncology, Mohammed V University Medical School, Rabat, Morocco; 2 Surgical Oncology, Hospital El Farabi, Mohammed Ist University, Oujda, Morocco; 3 Surgery Department, Sidi Mohammed Ben Abdellah University, Fes, Morocco; 4 Surgery Department, Military Hospital, Rabat, Morocco; 5 Batna Anticancer Center Alger, Batna, Algeria; 6 Anatomopathology Department, National Institute of Oncology, Rabat, Morocco; 7 Surgical Department “A”, Ibn Sina Hospital, Rabat, Morocco; 8 Private surgical oncology center, Salé, Morocco; 9 Surgical Department “C”, Ibn Sina Hospital, Mohammed V University. Medical School, Rabat, Morocco

**Keywords:** Rectal neoplasm, Neoadjuvant treatment, Complete pathological response, Disease-free survival, Predictive factors

## Abstract

**Background:**

Many data suggest that patients with low rectal adenocarcinoma who achieved ypT0N0 status have improved survival and disease-free survival (DFS) compared to all other stages however only few data are available regarding the specific prognosis factors of this subgroup. This study aimed to evaluate predictive factors for disease free survival after complete pathological response (CPR) in cases of low rectal adenocarcinoma.

**Materials and methods:**

From January 2005 to December 2013, all patients with low rectal adenocarcinoma who underwent neoadjuvant chemoradiotherapy followed by total mesorectal excision and achieved CPR were included at 7 Moroccan and 1 Algerian centres. Predictive factors for disease-free survival were analysed by uni and multivariate analysis.

**Results:**

Eigthy-four (12.1%) patients achieved a CPR (ypT0N0). Multivariate analysis revealed that both poorly differentiated tumors (OR, 9.23; 95 CI 1.35–62.82; *P* = 0.023) and the occurrence of perineal sepsis (OR, 13.51; 95 CI 1.96–93.12; *P* = 0.008) were independently associated with impaired DFS.

**Conclusions:**

Patients with low rectal cancer who exhibited a CPR after neoadjuvant therapy have good prognoses; however, the occurrence of perineal sepsis and/or poor initial differentiation may be associated with impaired DFS in these patients.

Trial registration: The study was retrospectively registered the 28th July 2018 in ClinicalTrials.gov register with the reference NCT03601689.

## Background

Currently, the standard treatment for mid-low locally advanced rectal cancer is neoadjuvant (chemo)-radiation therapy followed by curative surgical resection according to the guidelines of both the European Society of Medical Oncology (ESMO) and the National Comprehensive Cancer Network (NCCN) [1]. Most patients exhibit a substantial downstaging that may lead to complete pathological responses (CPRs) in 15 to 20% of cases, as defined by the absence of viable tumour cells after full pathologic examination of the resected specimen (ypT0N0), which is referred to as stage 0 disease [2]. These findings have helped many authors to reconsider the need for surgical resection after a CPR [3–6]; however, no definitive surrogate of a CPR (clinical, biological or radiological) has been reported in the literature, and surgical resection remains the standard treatment.

Although many data suggest that patients with ypT0N0 status have better prognoses and improved survival and disease-free survival (DFS) compared to all other stages, few data are available regarding the details of oncological outcomes [7, 8]. Additionally, little is known about the specific prognostic factors for this subgroup of patients.

The aim of this study was to evaluate the long-term oncologic outcomes and predictive factors for DFS after a CPR of low rectal adenocarcinoma.

## Methods

### Study design

This was a retrospective multicenter case series study conducted by the Moroccan Society of Surgery. Eight centres agreed to participate: seven were in Morocco (i.e., the Surgical Departments A and C, Ibn Sina Hospital, the National Institute of Oncology, the Military Hospital in Rabat, the Surgical Department B in Hassan 2 University Hospital in Fes, the Oncological Surgical Department in Oujda and a private oncological centre), and one was located in Algeria (the Anticancer Centre, Batna).

An online form (Google forms) was sent to each participating centre for data collection, and all information was anonymous. Each investigator obtained ethical approval from their own centre. This study was reviewed and approved by the Ethics Committee of the Ibn Sina Hospital (Rabat, Morocco). The study was registered in ClinicalTrials.gov register with the reference NCT03601689 and has been reported in line with the PROCESS criteria [9].

### Inclusion and exclusion criteria

The inclusion criteria were as follows: (1) patients over 18 years of age with a histologically proven low rectal adenocarcinoma, (2) no previous or synchronous colorectal disease, (3) UICC stage I-III patients who underwent neoadjuvant chemoradiotherapy, (4) chemotherapy followed by total mesorectal excision (TME), and (5) a CPR defined as ypT0N0.

The exclusion criteria were as follows: patients with metastatic tumour’s or missing data.

### Endpoints

This study primarily aimed to analyse the local and distant recurrence rates and secondarily aimed to determine the predictive factors of DFS.

### Therapeutic protocol

All patients from Moroccan centres underwent preoperative radiotherapy (50.4 Gy) and concomitant chemotherapy (Capecitabine 825 mg/mg twice daily). Patients in the Algerian centre were enrolled in a prospective study with a short protocol of 5 × 5 Gy radiotherapy. All patients underwent surgery at least 6–8 weeks after the end of neoadjuvant treatment.

Tumours up to 3 to 4 cm underwent an anterior resection with stapled colorectal or manual coloanal anastomosis, whereas smaller tumours with no invasion of the external sphincter underwent an intersphincteric resection. In cases with no distal margin or external sphincter involvement, an abdominoperineal resection (APR) was performed, followed by either an iliac colostomy or perineal pseudocontinent colostomy [10].

Because all selected patients achieved a CPR, no adjuvant treatments were administered, according to the guidelines.

### Pathological assessment

A CPR was defined as a pathological report of the surgical specimens describing a status of ypT0N0 according to the Dworak classification [11]. All other cases (ypN+ and/or ypT+) were considered non-responses and were excluded.

The specimens were analysed using very similar protocols in each institute (i.e., 5-mm slices of the rectal tumours were subjected to intensified evaluations of the tissue at the tumour site and at 2 to 3 sublevels in cases in which no tumour was found in the initial block). A second pathologist reviewed all CPR surgical specimens.

### Early postoperative outcomes

The early postoperative outcomes included the in-hospital and/or one-month postoperative periods. Complications were evaluated according to the Clavien-Dindo classification [11].

Perineal sepsis was defined as the presence of a postoperative clinical anastomotic fistula (pus or faecal discharge from the drain, pelvic abscess, peritonitis, recto-vaginal fistula, or discharge of pus from the rectum) in cases of colorectal or coloanal anastomoses and as perineal infection (i.e., the presence of a pelvic abscess or wound dehiscence) in cases of APR. [12]

### Assessment of oncologic outcomes

Patients were followed up alternately by a surgeon and an oncologist via a clinical examination, a stoma examination and a liver ultrasound or thoraco-abdominopelvic CT examination every three to 4 months for 2 years, every 6 months for 3 years after that, and once per year thereafter. A postoperative recurrence was defined by biopsy-proven or radiographic evidence of local or distant recurrent disease. DFS was defined as the period between the day of surgery and the date of recurrence or the last date of follow-up.

### Statistical analysis

Continuous variables are presented as the means ± SDs or as the medians with the interquartile ranges, and categorical variables are expressed as frequencies and percentages. SPSS software (SPSS 13.0; SPSS Inc., Chicago, IL) was used for the univariate and multivariate analyses that were applied to identify the predictive factors for recurrence in patients with ypT0N0 status.

Only patients with sufficient follow-up were included in the analysis of the predictive factors of DFS (patients who died postoperatively and those lost to follow up were excluded from this analysis). The analysed variables were age, sex, ASA score, distance from the anal verge, differentiation degree **at the initial rectal biopsy**, T and N pre-therapeutic stages, the type of neoadjuvant radiotherapy, the median interval between preoperative CRT completion and surgery, the type of surgical procedure and the occurrence of perineal sepsis. Comparisons between groups were performed using the χ2 test or Fisher’s exact test as appropriate. All variables associated with a poor functional result with a *P* value equal to or less than 0.1 in the univariate analysis were introduced into a multivariate logistic regression model that included the calculations of the ORs and 95% CIs. A P value of < 0.05 was considered statistically significant.

Survival was analysed according to the Kaplan-Meier method. The predictive factors of DFS were analysed by Cox regression.

## Results

From January 2005 to December 2013, 694 consecutive patients underwent neoadjuvant treatment followed by TME in the 8 centres.

Of these, 84 **(**12.1%) patients achieved a CPR (ypT0N0). The mean age of these patients was 54.5 years (SD 12 years). The demographic details and treatment modalities are provided in Tables 1 and 2.

**Table 1:** Demographics and surgical procedures in 84 patients with complete pathological response (CPR) after neoadjuvant treatment

Characteristics	N (%)
Gender	
Male	38 (45.2)
female	46 (54.8)
Mean age ± SD (years)	55,2 ± 12,5
ASA score	
1	48 (57)
2	13 (15.5)
Missing	23 (27.5)
Median distance from the anal verge (cm) (quartiles)	4 (3–6)
Histologic differentiation	
Well differentiated	56 (66.7)
Poorly differentiated	14 (16.7)
Missing	14 (16.7)
Pretherapeutic T stage	
T1-T2	19 (22)
T3- T4	52 (62)
missing	13 (15.5)
Pretherapeutic N stage	
N0	20 (23.8)
N1	53 (63.1)
Missing	11 (13.1)
Neoadjuvant radiotherapy	
Concomitant chemotherapy	76 (90.5)
45Gy	6 (7.2)
25 Gy	2 (2.5)
Median delay CRT/ Surgery (weeks)	7 (6–8)
Surgical approach	
Laparoscopy	26 (31)
Open procedure	58 (69)
Surgical procedures n(%)	
Anterior resection	51 (60.8)
Coloanal anastomosis	31 (37)
Colorectal anastomosis	20 (23.8)
APR	33 (39.3)
Left Iliac colostomy	23 (27.4)
Perineal pseudocontinent colostomy	10 (11.9)

**Table 2:** Thirty-days surgical outcomes and pathological details in 84 patients with complete pathological response (CPR) after neoadjuvant treatment

Thirty days surgical outcomes	N (%)
Mortality	3 (3.6)
Global complications	25 (29.8)
<IIIa	13 (15.5)
≥ IIIa	12 (14.3)
Perineal sepsis	16 (19)
Pathological details	
Median size of the scar cm (range)	2 (0–10)
Median Distal margins in cm (Range)	2.35 (0–8)
Median Circumferential margins in mm (range)	2 (0–20)
Median lymph node number n (range)	8 (0–22)
Acellular mucine n (%)	15 (18)

The 30-day mortality rate was 3.6%, and the global complication rate as defined by a Clavien-Dindo score (CD) ≥ IIIa was 14.3%. Perineal sepsis occurred in 16 patients (19%).

### Pathological results

The pathological examinations revealed that the median tumour scar was 2 cm with median distal and circumferential margins of 2.35 cm and 2 mm, respectively. Fifteen patients (18%) had residual acellular mucin, and the median number of retrieved lymph nodes was 8 (range 0–21).

### Oncologic outcomes

Two patients were lost to follow up, and 3 patients died postoperatively and were excluded from the oncologic analysis. The median follow-up duration was 30 months (range: 3 to 120 months).

Of the 79 patients, 9 patients developed recurrence (11.4%), including 4 local recurrences, 3 distant recurrences and 2 simultaneous local and distant recurrences.

### Predictors of disease-free survival

The univariate analysis indicated that poorly differentiated tumours (OR, 10.75;95 CI 1.90–58.67; *P* = 0.007) and the occurrence of perineal sepsis (OR, 7.32; 95 CI 1.81–29.50; *P* = 0.005) were significantly associated with recurrences. The other variables that were evaluated (i.e., age, sex, ASA score, distance from the anal verge, T and N pre-therapeutic stages, type of neoadjuvant radiotherapy, the median delay to CRT surgery and the type of surgical procedure) were not associated with impaired DFS. (Table 3).

**Table 3:** Univariate and multivariate analysis of predictive factors of impaired disease-free survival

	Univariate analysis		Multivariate analysis	
	HR (95% CI)	*P*	HR (95% CI)	*P*
Gender		0.79		
Male	1			
Female	1.2 (0.32–4,45)			
Histologic differentiation		0.007		0.02
Well differentiated	1		1	
Poorly differentiated	10.75 (1.90–58.67)		9.23 (1.36–62.82)	
Pretherapeutic T stage		0.94		
T1-T2	1			
T3-T4	0.94 (0.17–5.17)			
Pretherapeutic N stage		0.66		
N0	1			
N1	0.68 (0.12–3.72)			
Neoadjuvant treatment		0.66		
radiotherapy alone	1			
Chemoradiotherapy	0.61 (0.07–5.43)			
Surgical procedure		0.89		
APR	1			
Conservative intervention	1.1 (0.29–4.10)			
Perineal sepsis		0.005		0.008
No	1		1	
Yes	7.32 (1.81–29.50)		13.51(1.96–93.12)	

**Fig. 1:**
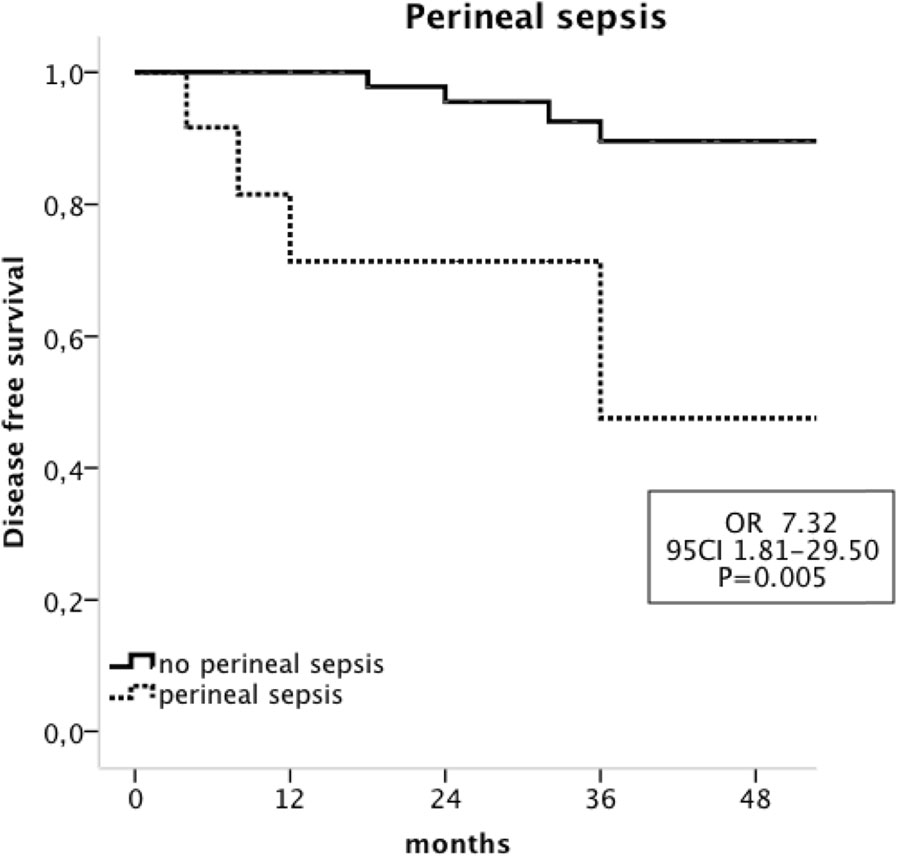
Kaplan-Meier analysis of Disease free survival in patients with CPR according to the occurrence of perineal sepsis

**Fig. 2:**
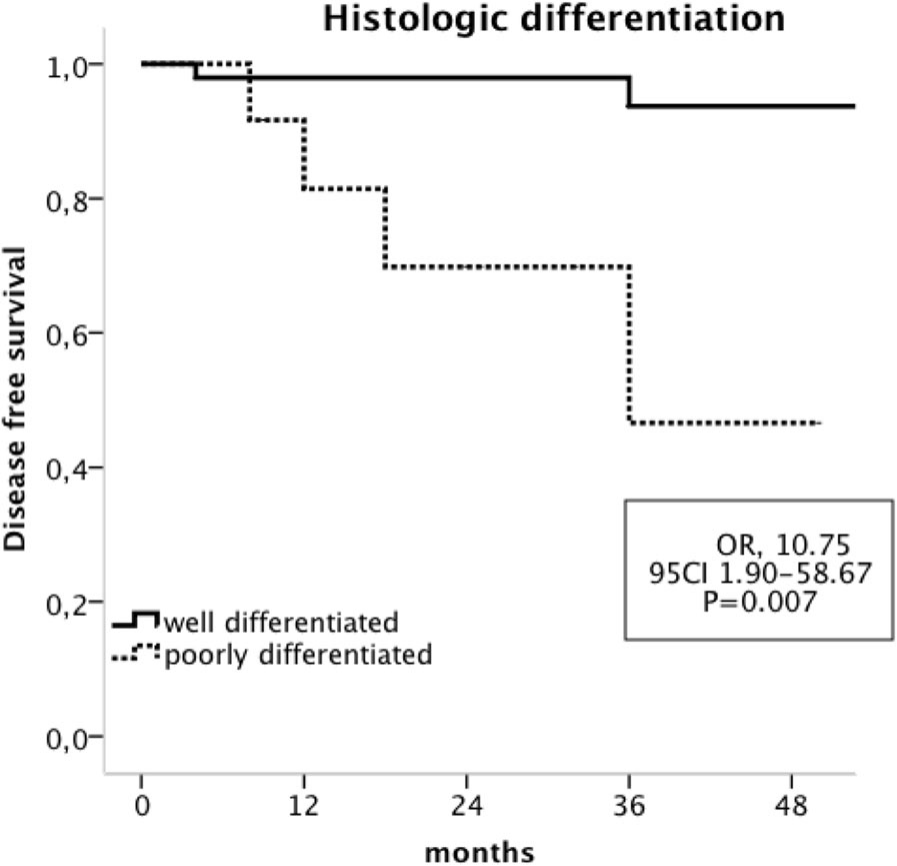
Kaplan-Meier analysis of Disease free survival in patients with CPR according to the pretherapeutic histologic differentiation

In the multivariate analysis, both poorly differentiated tumours (OR, 9.23; 95 CI 1.35–62.82; *P* = 0.023) and the occurrence of perineal sepsis (OR, 13.51; 95 CI 1.96–93.12; *P* = 0.008) were independently associated with impaired DFS. (Figs. 1 and 2).

## Discussion

This study demonstrated that for patients with a CPR after neoadjuvant treatment followed by TME, the recurrence rate was 12.6%. Both poorly differentiated tumours and the occurrence of perineal sepsis were factors that independently predicted impaired DFS in this population with ORs of 9.23 and 13.51, respectively.

Patients who develop a CPR after neoadjuvant treatment exhibit better prognoses, a reduced propensity for local or distant recurrence and improved survival [4, 13, 14]. However, one study reported that even for ypT0N0M0 low rectal cancers, after a median follow-up of 48 months, 61 of 419 patients developed recurrences, which resulted in a five-year DFS of 83% [4].

A CPR after chemoradiation therapy reduces, but does not eradicate, the risks of local and distant metastases [4, 6, 7]. Additionally, there are no data regarding the predictive factors of impaired DFS in this specific population of patients with good prognoses who achieved a CPR for low rectal adenocarcinoma.

Growing evidence and accumulating data indicate that anastomotic leakage (perineal sepsis) affects not only the short- but also the long-term survival [15]. In a recent meta-analysis, anastomotic leakage was associated with a greater rate of local recurrence (HR 1.71; 95% CI 1.22–2.38) and a decrease in overall survival (HR 1.67; 95% CI 1.19–2.35) [16]. A major explanation of this finding is that tumour cells are exfoliated from the primary tumour and seed the intraluminal local resection environment [17]. When perineal sepsis occurs, these cells may find an adequate environment for implementation and growth that leads to pelvic recurrence. In vitro studies have demonstrated a multifactorial mechanism by which inflammatory responses of the postoperative peritoneum and/or pelvic sepsis may enhance local recurrence, and this mechanism involves a combination of the amplification of angiogenesis and stimulation of both the migration and invasion capacities of tumour cells [18]. This is a plausible theory in cases of CPR because no viable tumours are found in the resection specimens, leading us to believe that unique viable tumours are left in the perirectal (or extra-fascial) environment and may be reactivated by postoperative local inflammation.

Poor tumour differentiation is one of the best-known predictive factors of local and distant recurrence [18]. It is also a predictive factor of an incomplete response to neoadjuvant chemoradiotherapy [19]. However, it remains unclear how poorly differentiated tumours (especially the SRCC subtype) enable aggressive outcomes even after a CPR [20, 21]. Linosilva et al. reported a case with the presence of microscopic clinical carcinomatosis implants with a total mural tumour response [21]. This finding is a reminder of the strong potential for SRCCs in other locations and the high probability of recurrence, especially for peritoneal carcinomatosis [22, 23].

Another theory of local and distant recurrence after a CPR may be that the tumour is left behind in the patient during surgery [4]. Lateral pelvic lymph node (LPLN) involvement is associated with poorer survival and a high rate of locoregional recurrence [24–26]. The management of this disease is completely different in Japan than in all other countries. In Western countries, LPLNs are considered metastatic and can only be managed by preoperative chemoradiotherapy combined with a standard TME, whereas in Japan, lateral pelvic lymph node dissection (LPLND) represents a standard regional lymphadenectomy. Akiyoshi et al. reported that preoperative CRT alone cannot eradicate LPLN involvement, but excellent local control and survival can be achieved with the combination of preoperative CRT and LPLND [27]. Because MRI before CRT seems to be useful for predicting LPLN metastases, extended lymphadenectomy may be indicated for selected patients with advanced low rectal cancer with preoperative cN status on a preoperative MRI [28] to avoid recurrence, even after a CPR.

Some limitations should be considered when interpreting our study results, including the retrospective multicenter study design, limited follow-up period, small size of the studied population and potential bias inherent in the data collection and analysis. We acknowledge that deficiencies in the perioperative evaluations may exist, such as the lack of systematic post-CRT MRI examinations, differences in radiotherapy protocols between centres, and difficulties in the pathological analysis of the specimens in cases in which no tumour was found. However, very specific attention was given to all specimens with a pCR, and they were reviewed by a second pathologist according to a standardized protocol.

To our knowledge, this is the first study to suggest that even in the group with the best prognoses and CPR after chemoradiotherapy for low rectal cancer, the occurrence of perineal sepsis and/or poor initial differentiation may be associated with impaired DFS.

Based on these findings, other options for the management of advanced low rectal cancer may be discussed, such as LPLND in patients with pre-treatment cN lPLN status on MRI results and the addition of oxaliplatin to fluorouracil-based neoadjuvant chemoradiotherapy and adjuvant chemotherapy to reduce the risks of both local and distant recurrences [29].

## Conclusion

This study demonstrated that a CPR is associated with a 12.6% rate of recurrence and that perineal sepsis and histologically poor differentiation are associated with worse DFS times among patients with a CPR. Additional aggressive therapeutic options should be considered when these risk factors are present.

## Data Availability

The datasets used and/or analysed during the current study are available from the corresponding author on reasonable request.

## Abbreviations

APR: Abdominoperineal resection
ASA score: American Society of Anesthesiologist
CD score: Clavien Dindo score
CI: Confidence interval
cN: Clinical Node
CPR: Complete pathological response
CRT: Chemoradiotherapy
DFS: Disease-free survival
ESMO: European Society of Medical Oncology
HR: Hazard Ratio
LPLN: Lateral pelvic lymph node
MRI: Magnetic Resonance Imaging
NCCN: National Comprehensive Cancer Network
OR: Ods ratio
SD: Standard Deviation
SRCC: Signet Ring Cancer Cell
TME: Total Mesorectal Excision
UICC: Union Internationale Contre le Cancer
